# What links to psychological needs satisfaction and excessive WeChat use? The mediating role of anxiety, depression and WeChat use intensity

**DOI:** 10.1186/s40359-021-00604-8

**Published:** 2021-07-13

**Authors:** Qiufeng Gao, Yanzhen Li, Ziwei Zhu, En Fu, Xiangyu Bu, Shan Peng, Yanhui Xiang

**Affiliations:** 1 Department of Sociology, Law school, Shenzhen University, Shenzhen, China; 2 Department of Psychology, Hunan Normal University, Changsha, China

**Keywords:** Psychological needs satisfaction, Anxiety, Depression, WeChat use intensity, Excessive WeChat use

## Abstract

**Background:**

Excessive online social network sites (SNSs) use, such as Facebook or WeChat overuse, has become a severe problem and have caused negative consequences. It is especially important to examine what causes excessive WeChat use in the Chinese population. This study explored the critical role of affective states and WeChat use intensity in the relationship between psychological needs satisfaction and excessive WeChat use based on the self-determination theory and the emotional motivation theory.

**Methods:**

952 Chinese college students aged 18 to 25 completed an online survey that measured psychological needs satisfaction, depression, anxiety, WeChat use intensity, and excessive WeChat use.

**Results:**

Path analysis demonstrated that anxiety, depression, and WeChat use intensity mediated the effect of psychological needs satisfaction on excessive WeChat use. More importantly, the chain mediation model indicated that psychological needs satisfaction could influence excessive WeChat use through the “anxiety—WeChat use intensity” path, but not the “depression—WeChat use intensity” path.

**Conclusion:**

The current study could not only contribute to theoretical development, but also guide mental health practice by showing that improving psychological needs satisfaction may restrain excessive WeChat use through regulating affective states and Wechat use intensity.

## Background

With the prevalence of technology today, social network sites (SNSs) such as Facebook, WhatsApp, and Line are playing indispensable roles in people's daily lives. In China, WeChat is the most important and popular media that provides SNS functions, especially for youth [1]. Individual can obtain supportive social interactions [2], fast messaging, mobile payment services and all kinds of life necessary small procedures [3] quickly through WeChat. During the first quarter of 2019, the monthly active users of WeChat reached 1112 million [4] and 18 to 24 year olds have become the main user group for social networking [1].

Alongside the convenience that SNSs has brought to our social lives, SNSs excessive use has become a severe problem and have caused negative consequences [5] such as cognitive-emotional preoccupation [6], envy [7], depression [8], and addictive behaviors [9]. As excessive WeChat use tendency has become a serious social problem in China, especially for college students, who just have less limited access to their mobile phones and lack enough self-control [10, 11]. Moreover, social life is another main task except learning at this special stage [12, 13]. Wechat, as one of the most popular use social media to meet their basic psychology and reality life needs, has integrated into theirs’ lives. Thus, it is especially important to examine its trigger factors and underlying mechanisms and help them to reduce the negative consequences caused by excessive WeChat use.

Previous studies on excessive SNSs use suggest that basic psychological needs satisfaction may be an important trigger factor for youths’ excessive SNSs use [14, 15]. According to self-determination theory [16], basic psychological needs are fundamental and essential “nutriments” for individual development [16, 17]. Human usually seek to satisfy the following three basic, inner psychological needs: (1) competence, (2) autonomy, and (3) relatedness. High satisfaction of these inner psychological needs is strongly linked with psychological well-being and promote individuals effective self-regulation [17]. However low psychological needs satisfaction can trigger internalizing psychological problems, such as high anxiety and depression, and externalizing behavior problems, including addictive behaviors such as Internet addiction, and SNSs addiction [18–20]. In accordance with this theory, many studies suggest that psychological needs satisfaction could negatively predict individual excessive SNSs use [21–23]. For example, Ryan et al. [21] suggests that media can provide users with specific gratifications which may compensate for lower need satisfaction [21]. Another study by Sheldon et al. [22] similarly showed Low levels of relatedness in daily life seem to link with increased Facebook usage [22]. Moreover, Reinecke et al. [23] notes that, in addition to relatedness need satisfaction, the intrinsic needs for competence and autonomy can also be satisfied by Facebook usage [23].

**Fig. 1:**
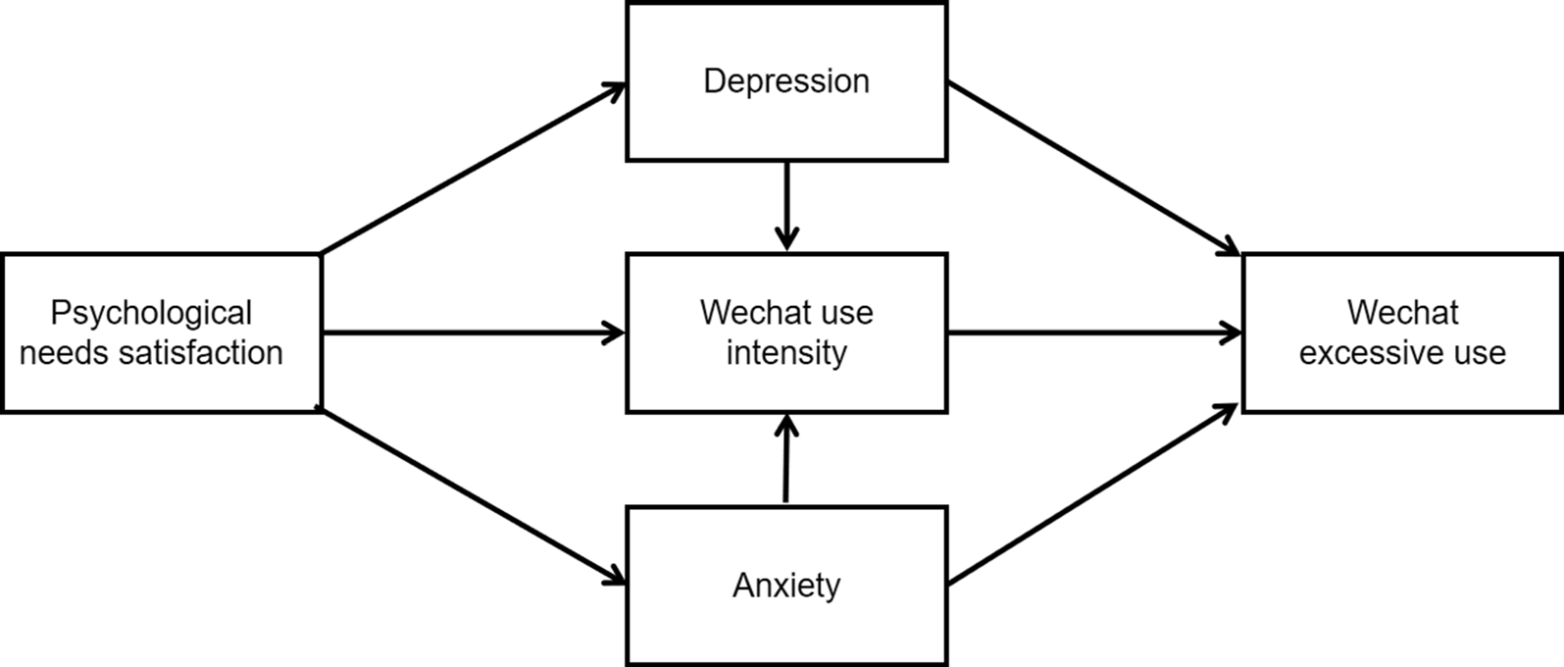
The assumed chain mediation model

Although existing previous studies have demonstrated the direct association between psychological needs satisfaction and excessive SNSs use, fewer studies have focused on its underlying mechanisms. Only a few studies highlighted the underlying mechanisms between psychological needs satisfaction and SNSs use [15, 24]. For example, Feng et al. [24] explored the mediating role of the role of cognitive and the moderating role of self-regulation in the association between Chinese college students’ psychological needs and internet interaction [24]. Therefore, from the perspective of the self–determination theory [16] and the emotional motivation theory, the present study aims to explore the mediating role of negative affective states and WeChat Use Intensity in the association psychological needs satisfaction and excessive WeChat use among Chinese college students (See the conceptual model in Fig. 1). These findings could not only improve our knowledge of how psychological needs satisfaction lead to excessive WeChat use of young users, but also guide the development of prevention and intervention strategies to protect college students from excessive WeChat use.

### The mediating roles of negative affective states

Negative affective states is usually an unpleasant or unhappy affective states evoked in individuals to express a negative affect towards an event or person, which is not satisfied with personal expectations or needs, usually including anxiety, depression, anger, and loneliness[25]. In the present study, we mainly focused on two negative affective states, anxiety and depression, which are not clinical variables, just anxiety and depression symptoms of individuals, for the following two reason: First, there are an increasing incidence of anxiety and depression among college students in recent years [26, 27]. One study in Chinese found that nearly half of the college students had moderate levels of mental health concerns, including anxiety and depression [28]. Second, Previous studies found that excessive use of SNSs mainly linked to depression [29–32] and anxiety [33, 34]. Specifically, people with high anxiety or depression are often uncomfortable with face-to-face communication and face much more interpersonal difficulties [35–37], and would spend more time to take online communication as compensation [38].

Besides, negative affective states could be evoked by unsatisfied individuals’ basic intrinsic needs. Some studies have proved that low satisfaction with basic psychological needs was related to negative emotions and anxiety symptoms [39, 40]. Negative emotions arise from unmet psychological needs, which drive external behavioral performance [41, 42]. This is consistent with the emotional motivation theory, which asserts that motivation is one of the main functional attributes of emotion. Emotional motivation can stimulate behavior by amplifying internal drive [43–45]. In SNS usage, emotional motivation theory seems to confirm that the internal drive of psychological needs may be amplified through emotional motivation and affect the use of SNS. Self-determination theory also posits that people can maintain optimal functions and achieve positive personal growth by satisfying basic needs [16]. When the basic needs are hindered, individuals with unmet basic needs will fall into a maladaptive mode and then produce negative affective states or emotions. These individual needs may temporarily alleviate the negative emotions caused by the frustration of needs by pursuing nihilistic external goals or compensatory behaviors. This is why when the basic psychological need is unsatisfied and individuals may lead to overuse of WeChat to alleviate negative affective states.

Previous studies had suggested that the use patterns of SNS in individuals with depression and anxiety disorder were different [46]. Depression and anxiety could lead to different content and frequency of SNS use [47–49]. Therefore, we hypothesized two common negative affective states, depression and anxiety, might have played essential roles in linking psychological needs satisfaction and excessive WeChat use.

### The mediating roles of WeChat use intensity

SNS use intensity is determined by the number of friends in SNS, the time spent on SNS, and the frequency of accessing and using SNS [50–52]. Ellisonand colleagues proposed that Facebook use intensity could be measured by the users’ participation activities, such as Facebook activities, the number of Facebook "friends", and the average daily use time [52]. Similarly, Salehan and Negahban [51] revised Ellison and colleagues’ measurement [52] and developed a tool to measure emotional ties to WeChat and the degree of WeChat integration into daily life. It is worth noticing that Salehan and Negahban’s measurement did not involve problematic consequences.

There are substantial differences between WeChat use intensity and excessive WeChat use. The key points include that first, from a purely definitional perspective, there are different definitions between excessive WeChat use and WeChat use intensity. Excessive WeChat use refers to the performance on the psychological level, and emphasizes the degree of mood modification, salience, conflict and other psychological symptoms caused by WeChat use. However, WeChat use intensity usually emphasizes the degree of use at the behavioral level, and mainly evaluates the daily situation of individuals using WeChat, such as the number of friends in WeChat, the length of time spent on WeChat, the frequency of accessing WeChat and other indicators; Second, from the perspective of the relationship between two variables, WeChat use intensity is an indicator of excessive WeChat use. Salehan and Negahban put forward that SNS intensity was a significant predictor of SNS and mobile addiction [51]. Van Deursen and colleagues also believed that frequent smartphone use was an important contributor to addictive smartphone use behaviors [53]. Previous studies had also suggest that the frequency of technology use has been demonstrated as a predictor of problematic use, such as increased frequent SNS use can cause problematic SNS use [51, 53, 54]. Excessive WeChat use is one of problematic SNS use, it can be inferred that WeChat use intensity may be a predictor of Excessive WeChat use. Besides, WeChat use intensity involves some items or variables about usage status, such as the spent time, the participation degree, and the frequency of WeChat use. Empirical studies used these variables as independent variables to explore their impact on excessive WeChat use [50, 55, 56]. They proved that WeChat use intensity (e.g.the spent time; the frequency; the participation degree) could significantly predict excessive WeChat use. The results of these research provided a basis for explore the impact of WeChat use intensity on excessive WeChat use. Based on this, we hypothesized that WeChat use intensity might play an important role in the relationship between psychological needs satisfaction and excessive WeChat use.

WeChat use intensity and excessive WeChat use are often affected by negative affective states, which can drive behavioral problems. The frequency of technology use acts as a mediator between psychopathology such as depression and anxiety and problematic technology use [53, 57]. Negative emotion may reduce or increase SNS activity frequency [48] such that SNS excessive use such as excessive WeChat use is more likely to appear [58]. At the same time, psychological needs satisfaction will also affect the strength of WeChat use. Motivation for media use (such as information acquisition, social interaction, etc.) change the frequency, number and intensity of media use [59–61], and motivate users to engage in specific social media usage behaviors [62, 63]. Therefore, WeChat use intensity itself may cause excessive WeChat use. Based on this, we hypothesized that psychological needs satisfaction dould predict excessive WeChat use through the chain mediation of “negative affective states—WeChat use intensity”.

### The present study

In summary, based on the self-determination theory and emotional motivation theory, this study explored the effect of psychological needs satisfaction on excessive WeChat use and the underlying mechanism of negative affective states. This study would be the first to investigate the mechanism affecting excessive WeChat use from a more holistic perspective of the concept of psychological needs satisfaction, using the self-determination theory and emotional motivation theory.

This study explored the relationship between psychological needs satisfaction and excessive WeChat use as well as the psychological mechanism underlying their relationship. Four hypotheses were tested in this study:

#### **H1**

Based on the self-determination theory, Low psychological needs satisfaction could lead to excessive WeChat use.

#### **H2**

Based on the emotional motivation theory, negative affective states could mediate the relationship between psychological needs satisfaction and excessive WeChat use.

#### **H3**

WeChat use intensity could mediate the relationship between psychological needs satisfaction and excessive WeChat use directly.

#### **H4**

The chain mediation of “negative affective states—WeChat use intensity” could affect the influence of psychological needs satisfaction on excessive WeChat use.

## Method

### Participants and procedure

In total, 952 college students with smartphones from a comprehensive university in ShenZhen, China were invited to participate in the current study. One-hundred-and-three participants were excluded from analyses due to displaying a continuous response time of more than 10 min or less than 1 min, having the same answer consecutively for more than 20% of total question items. Data from 849 participants were used for modelling. Covariates controlled in the current study included demographic information (e.g. age and gender) and indicators of daily WeChat use (e.g. daily WeChat use time). Four-hundred-and-fifty-one participants were male (53.1%) and the mean (SD) age was 19.0 (1.36) years old. Majority of the participants used WeChat for 1 to 3 h daily (see Table 1 for details).

**Table 1:** Sample characteristics and covariates

Variables	*n*	%	M (SD)
Age	849		19.0 (1.36)
*Gender*			
Male	451	53.1	
Female	398	46.9	
*The average time of SNS use (e.g., WeChat) per day*			
< 1 h	126	14.8	
1–3 h	416	49.0	
3–5 h	186	21.9	
5–7 h	68	8.0	
> 7 h	53	6.2	

*SNS* social network sites; *N* = 849; *M* mean; *SD* standard deviations; *h* hour

Institutional Review Board at the first author’s institution approved the current study procedure. Study participants were recruited from different colleges but took the same course. Trained research assistants visited the classes after obtaining teachers' consent. The assistants informed all participants that participation in this study was voluntary, and all data would be kept anonymous. Participants understood the confidentiality of their participation. Participants logged into a survey website (https://www.wjx.cn/) and filled a questionnaire that included measurements of psychological needs satisfaction, affective symptoms, excessive WeChat use, and WeChat use intensity. Participants spent 20 min on average to complete the survey. Previous studies had proved the effectiveness of online data collection [64].

### Measures

#### Psychological needs satisfaction

Twenty-one items from the Chinese version [65] of the Psychological Needs Satisfaction Scales by Deci and colleagues [66] were used to assess the level of psychological needs satisfaction. This Scale consisted of three subscales: (1) Autonomy needs were measured with seven items (e.g., “I feel like I can pretty much be myself in my daily situations.”), (2) Competence needs were measured with six items (e.g., “I have been able to learn interesting new skills recently.”), and (3) Relatedness needs were measured with eight items (e.g., “People are generally pretty friendly towards me.”). With total scores ranging from 5 to 105, higher scores indicated a higher level of satisfaction of psychological needs. Each item was measured on a 5-point Likert scale ranging from 1 (strongly disagree) to 5 (strongly agree). Higher scores on the scales represented a greater level of psychological needs satisfaction. Existing research has shown satisfactory reliability and validity of the Psychological Needs Satisfaction Scale in the Chinese population [67]. Cronbach’s alpha coefficient of the scales was 0.86, Cronbach’s alpha coefficient of three subscales were: Autonomy, α = 0.65; Competence, α = 0.61; Relatedness, α = 0.76.

#### Anxiety and depression

Anxiety and depression were measured by the Chinese version [68] of the Depression-Anxiety-Stress Scale (DASS-21) revised by Beck and Steer [69]. The Chinese version of the scale includes 21 items and 3 subscales: Anxiety (7 items), Depression (7 items), Stress (7 items). Each item was measured on a 4-point Likert scale ranging from 1 (never) to 4 (always)”. Higher total scores indicated stronger presence and higher levels of symptoms. The scale showed good discrimination validity so that could overcome the difficulty of distinguishing depression, anxiety, and stress due to comorbidity in previous clinical and empirical studies [70], thus previous studies had applied the scales extensively [49]. Cronbach’s alpha coefficient of three subscales were: Anxiety, α = 0.809; Depression, α = 0.863; Stress, α = 0.848. In current study, we focused on anxiety and depression subscales (14 items in total).

#### WeChat use intensity

The 5-item WeChat Use Intensity Scale was administered to assess participants’ frequency and intensity of WeChat usage. This scale was developed based on a previously validated scale [51] and included items such as “visiting SNSs is part of my everyday activity”. The original scale was modified to reflect participants’ intensity of using WeChat instead of general SNSs. Participants were instructed to rate items on a 7-point Likert scale ranging from 1(strongly disagree) to 7(strongly agree). The total score ranged from 5 to 35. Higher scores of WeChat use intensity represent intenser WeChat use. Cronbach's α in the current study was 0.860.

#### Excessive WeChat use

To measure the extent of excessive WeChat use, we used the Excessive WeChat Use scale developed by Hou and colleagues [71]. This 10-item scale measured mood modification, salience, and conflict. Each item was measured on a 5-point Likert scale ranged from 1 (rarely) to 5 (always). An example of an item was: ‘‘There were times when I would rather play on WeChat than go out with my friends’’. The total score ranged from 10 to 50. Higher scores on the Excessive WeChat Use scale represent severer excessive WeChat use. In our study, Cronbach's α coefficient of the scales was 0.890.

### Data analyses

SPSS 22.0 was used for data organization and data mining. The missing data, outliers, and normality were examined. Two steps were taken for data analyses. Firstly, data standardization was used to solve the collinearity problem. Subsequently, path analyses were conducted in Mplus 7.0 with ML estimator to test the mediating effect of depression, anxiety, and WeChat use intensity in the link between psychological needs satisfaction and excessive WeChat use.

The bootstrapping method produced 95% bias-corrected confidence intervals for the mediating effect by resampling 1000 samples to fit the data of excessive WeChat use. The method could obtain robust standard errors for parameter estimation and has greater statistical power on testing mediating effect [72]. If the 95% bias-corrected confidence interval for the parameter estimate does not contain zero, then the mediating effect are significant at α = 0.05 [73]. Model fit was estimated using five indices: The Chi-square statistics, the comparative fit index (CFI), the Tucker–Lewis index (TLI), the root mean square error of approximation (RMSEA), and the standardized root mean square residual (SRMR) [74]. Besides, the variances of all variables were fixed by a standardized solution (i.e., the STDYX option in Mplus) [75].

## Results

### Descriptive statistics and correlation

Preliminary analyses were performed to present univariate statistics and bivariate correlations among continuous variables in the study. As shown in Table 2, excessive WeChat use was negatively associated with psychological needs satisfaction (*r* =  − 0.159, *p* < 0.001), positively associated with anxiety (*r* = 0.205, *p* < 0.001), positively associated with depression (*r* = 0.184, *p* < 0.001), and positively associated with WeChat use intensity (*r* = 0.515, *p* < 0.001). In addition, psychological needs satisfaction was negatively associated with anxiety (*r* = − 0.423 *p* < 0.001) and depression (*r* = − 0.540, *p* < 0.001), which was in turn associated with WeChat use intensity (*r* = 0.155, *p* < 0.001). Finally, anxiety was positively associated with depression (*r* = 0.657, *p* < 0.001) as well as WeChat use intensity (*r* = 0.194, *p* < 0.01). However, there was no statistically significant correlation between depression and WeChat use intensity (*r* = − 0.013, *p* = 0.791).

**Table 2:** Descriptive statistics and correlations between variables in the study

Variables	1	2	3	4	5
1. Psychological needs satisfaction	1.000				
2. Anxiety	− 0.423^***^	1.000			
3. Depression	− 0.540^***^	0.657^***^	1.000		
4. WeChat use intensity	0.155^***^	0.194^***^	− 0.013	1.000	
5. Excessive WeChat use	− 0.159^***^	0.205^***^	0.184^***^	0.515^***^	1.000
*M*	3.46	1.67	1.46	5.09	2.54
*SD*	0.42	0.48	0.49	1.31	0.76

*N* = 849; *M* mean; *SD* standard deviations

*p < .05, **p < .01, ***p < .001

### Model fit

Based on previous research summarized above, we constructed a structural equation model for the current study (see Fig. 1). Based on the recommendations of Hu and Bentler [74], the following cutoff values were used to evaluate fit indices: RMSEA < 0.08, SRMR < 0.08, CFI > 0.90, and TLI > 0.05. The Chi-Square Test of Model Fit was statistically significant at the 0.05 level, χ^2^ (10) = 35.080 (*p* = 0.001) [75], which indicated a poor fit of the model to the data. However, according to Bryant’s [76] research, the judgment of the Chi-Square value on the model fit is related to the sample size. There may be inaccuracy in judging the suitability of the model fit based on Chi-Square value [76]. The other four indices also suggested a reasonably good fit of the proposed model, CFI = 0.983, TLI = 0.963, SRMR = 0.035, and RMSEA = 0.054 (90% CI = [0.035, 0.074]).

### Path analysis results

**Fig. 2:**
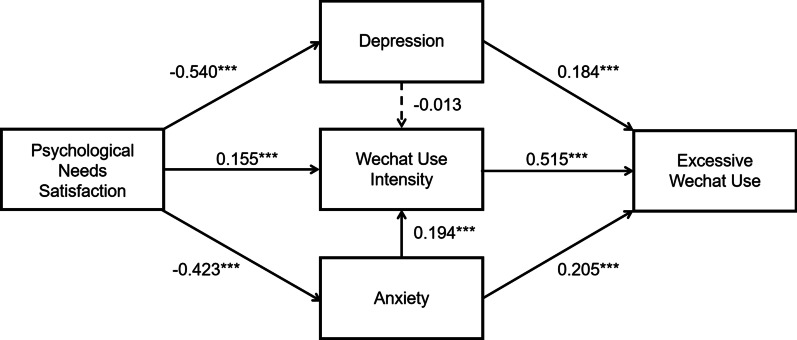
Path diagram and standardized parameter estimates for the final mediation model (N = 849). Note: *p < .05, **p < .01, ***p < .001

The assumed chain mediation model (Fig. 1) with three mediators (anxiety, depression and WeChat use intensity) revealed a good fit to the data (see Table 3 for details). The relationship between psychological needs satisfaction and excessive WeChat use was uniquely mediated by anxiety (95% CI = [− 0.120, − 0.052]), depression (95% CI = [− 0.140, − 0.055]), and WeChat use intensity (95% CI = [0.040, 0.122]). In addition, two chain mediation paths: “anxiety—WeChat use intensity” and “depression—WeChat use intensity” in the multiple mediation model were examined at the same time. It was of particular importance that the chain mediating effect of “anxiety—WeChat use intensity” (95% CI = [− 0.063, − 0.023]) was significant in the impact of psychological needs satisfaction on excessive WeChat use. However, the chain mediation path of “depression—WeChat use intensity” (95% CI = [− 0.023, 0.030]) was not significant (see Table 3). Therefore, our hypothesis about “psychological needs satisfaction—anxiety—WeChat use intensity—excessive WeChat use” was supported, while the other chain mediation hypothesis about “psychological needs satisfaction—depression—WeChat use intensity—excessive WeChat use” was not supported. Finally, coefficient segments in the “psychological needs satisfaction—depression—WeChat use intensity—excessive WeChat use” path were significant (see Figs. 1 and 2).

**Table 3:** Standardized indirect effects and 95% confidence intervals

Model pathways	Estimated	95%CI	
		Lower	Upper
PNS → Anxiety → EWU	− 0.087^a^	− 0.120	− 0.052
PNS → Depression → EWU	− 0.099^a^	− 0.140	− 0.055
PNS → WUI → EWU	0.080^a^	0.040	0.122
PNS → Anxiety → WUI → EWU	− 0.042^a^	− 0.063	− 0.023
PNS → Depression → WUI → EWU	0.004^a^	− 0.023	0.030

*PNS* psychological needs satisfaction; *WUI* WeChat use intensity; *EWU* excessive WeChat use

^a^Empirical 95% confidence interval does not overlap with zero

## Discussion

The purpose of this study was to reveal the relationship between psychological needs satisfaction and excessive WeChat use and of the underlying mechanism involving depression, anxiety, and WeChat use intensity based on the self–determination theory and the emotional motivation theory. Our findings showed that psychological needs satisfaction can directly affect excessive WeChat use, which verifies the view of self-determination theory that failure to meet the internal psychological needs may lead to problematic external behavior. More importantly, depression and anxiety played mediator roles in the link between psychological needs satisfaction and excessive WeChat use, validating the view of emotional motivation theory that internal psychological needs may be amplified through the emotional process, and then affect the external behavior. In addition, the final model showed that psychological needs satisfaction could influence excessive WeChat use through the chain mediation effect of "anxiety-WeChat use intensity". In conclusion, the current study demonstrated that psychological needs satisfaction could predict excessive WeChat use, and shed light on an important mechanism underlying excessive WeChat use: psychological needs satisfaction could influence excessive WeChat use via negative affective states and WeChat use intensity.

First, we validated the correlation between psychological needs satisfaction and excessive WeChat use in Chinese college students. Low satisfaction of psychological needs may lead to the problem of excessive WeChat use. This finding is consistent with the view of self-determination theory that low satisfaction of psychological needs may increase the incidence of in vitro addictive or problematic behaviors. The findings of current research supported the self-determination theory as well as its applicability in WeChat usage. The possible explanation for this result is that individuals with low levels of satisfaction of psychological needs usually fail to obtain sufficient happiness or effective self-regulation [17]. Low psychological needs satisfaction may trigger externalizing excessive SNSs use problems. Unmet psychological needs may prompt people to seek need satisfaction (e.g., social interaction and entertainment needs) through frequent SNS usage [24, 77]. The impact of psychological needs on individual mental and behavioral development has been extensively discussed [78]. Previous studies have shown that psychological need satisfaction is closely related to problematic behaviors such as substance abuse [79]. At the same time, previous studies have found that psychological needs such as perceived experience, achievement and control, attribution, interpersonal communication, escaping from reality and emotional coping, self-realization and self-transcendence are related to Internet addiction [80]. WeChat usage can compensate for the lack of needs satisfaction to some extent [24, 55].

Consistent with our second hypothesis, negative affective states played mediator roles between psychological needs satisfaction and excessive WeChat use. Tomkins’s emotional motivation theory held that the internal drive of psychological needs is amplified through emotional process, which could then stimulate action [43–45]. According to the emotional motivation theory, unmet psychological needs can trigger negative affective states such as anxiety and depression [39], the motivation to alleviate will subsequently induce internet-related addiction [80]. SNSs provide a channel for emotional self-expression and social connections [81, 82], which can help users to meet the basic needs of autonomy, competence, and relationships through excessive SNS use. Based on Tomkins's emotional motivation theory, unmet psychological needs can lead to negative affective states, which then prompt people to use WeChat pathologically, resulting in excessive WeChat use. This possible explanation can be given for the mediating effect of negative affective states between psychological needs satisfaction and excessive WeChat use. The findings of present research validated Tomkins's theory of emotional motivation in the population of Chinese college students.

Regarding the third hypothesis, psychological needs satisfaction can affect excessive WeChat use through both negative affective states and WeChat use intensity. The current study explored, for the first time, the relationships among satisfaction of psychological needs satisfaction, WeChat use intensity, and excessive WeChat use.The scattered existing research suggested that these three variables are associated with each other. Users may compensate for unmet needs and desires by increasing WeChat use intensity [59, 60, 63]. At the same time, excessive WeChat use may appear when the intensity of WeChat use for social and entertainment functions reaches the threshold of producing behavioral and psychological symptoms [50, 55, 56]. More noteworthy is that the influence of psychological needs satisfaction on excessive WeChat use could be explained by the chain mediated effect of "anxiety and intensity of use" but cannot be explained by the "depression—WeChat use intensity" chain mediation. Results of previous studies on the relationship between SNS usage and negative affective states were ambiguous. For example, there were evidences that depression and anxiety might have the same or different effects on SNS usage [46, 83]. The present study verified the different effects of anxiety and depression on excessive WeChat use. Chinese college students with low perceived psychological needs satisfaction experienced more depression and anxiety than their peers with high satisfaction. The heightened anxiety will then render these individuals vulnerable to excessive WeChat use through elevated frequency, duration, and scope of WeChat usage. Although the results did not show a "depression—WeChat use intensity" chain mediation effect, depression could lead to excessive WeChat use directly. This may be due to the essential differences in the external behavior of depression and anxiety [46]. There are different patterns and frequencies of SNS use among people with depression and anxiety [46, 47]. Anxiety promotes radical or aggressive behavior, and adolescents with high anxiety may choose to disclose their feelings more on SNS than in the real world [49, 84, 85]. On the contrary, depression impairs normal social activities while reducing access to and use of SNS [49, 84–86]. However, depression may increase psychological dependence on WeChat through other unknown variables, which can directly lead to excessive WeChat use.

The final model showed that the degree of psychological needs satisfaction could affect excessive WeChat use through anxiety, depression, WeChat use intensity, and the chain mediation mechanism of "anxiety—WeChat use intensity". For reference, in addition to think highly of the possible impact of psychological needs satisfaction on excessive WeChat use, we also need to pay more attention to negative affective states (anxiety and depression) and maintain a reasonable frequency of WeChat usage to deal with the problem of excessive WeChat use caused by unmet psychological needs. According to the findings, promoting needs satisfaction is important to prevent negative affective states (depression and anxiety) and to reduce excessive WeChat use. For people encounter the problem of excessive WeChat use, especially Chinese college students, they can try to improve the degree of psychological needs satisfaction or combine with appropriate emotional strategies to reduce the negative affective state (depression and anxiety) caused by unsatisfied psychological needs, so that the incidence of excessive WeChat use may decrease. Furthermore, findings from the current study can be considered to migrate to excessive use of other SNSs, and provide reference for future research on SNSs overuse.

## Limitation

Despite its innovative perspectives and contributions, the current study had several limitations. First, due to the scope of the current study, we averaged the item scores to represent psychological needs satisfaction. Future research may consider dividing the psychological needs satisfaction into three sub-dimensions for in-depth study. Second, the data were collected through self-report surveys thus warrant subjective biases. Multiple evaluation methods should be adopted to reduce such bias. Third, the cross-sectional nature of the current study warranted its limitation in generating causal inferences. Future research should adopt other research methods (E.g., longitudinal studies) to establish possible causal relationships. Finally, the conclusions were based on a group of Chinese college students thus might not be able to generate to other populations. Future research can invite groups from different cultural backgrounds as research objects to enhance the ecological validity of the results.

## Conclusion

Based on the self–determination theory and emotional motivation theory, the current study made the first attempt to investigate the possible association and underlying mechanisms between psychological needs satisfaction and excessive WeChat use from the perspective of negative affective states and WeChat use intensity. The current study not only validated the applicability of the self–determination theory and the emotional motivation theory to SNS use but also hinted on intervention practices for Chinese youth. For instance, handling psychological needs through emotional adjustment strategies can reduce users' desire for frequent WeChat usage and dependency.

## Data Availability

The datasets used and/or analysed during the current study are available from the corresponding author on reasonable request.

## Abbreviations

SNSs: Social network sites
RMSEA: Root mean square error of approximation
SRMR: Standardized root mean square residual
CFI: Comparative fit index
TLI: Tucker–Lewis index
